# The predictive–affective loop for directors: a target-state design framework for audiovisual directing

**DOI:** 10.3389/fpsyg.2026.1760222

**Published:** 2026-07-01

**Authors:** Youngtaek Jeong

**Affiliations:** Independent Researcher, Incheon, Republic of Korea

**Keywords:** appraisal, emotion, event segmentation, film directing, media psychology, PAL-D, predictive processing, sensory density

## Abstract

In this Hypothesis and Theory article, we propose the PAL-D (Predictive–Affective Loop for Directors) framework as a mid-level model linking director-side design decisions to the viewer’s predictive–affective response in film and television. Building on predictive processing, event segmentation research, and research on emotion and appraisal, we first lay out the viewer-side PAL (Predictive–Affective Loop) model, which characterizes viewing as a hierarchical Gaze–Prediction–Appraisal–Integration architecture in which prediction error, physiological arousal, appraisal, the prevailing affective condition, and an integrated episode-level affective condition interact over time. We then introduce PAL-D as a director-side grid that proposes four representative target-state patterns—calm, tension, crisis, and relief—four higher-order design axes (sensory density, framing and focus, narrative–contextual cueing, and affective–empathic cueing), and six perceptual–formal levers (camera position and distance, camera movement, framing and composition, light and color, editing rhythm and transitions, and sound and music). PAL-D treats the time course of prediction error as the primary organizing dimension and treats each target state as an intended trajectory of prediction error with characteristic arousal and affective profiles, against which empirically observed trajectories can later be compared. Rather than a fully quantified model, PAL-D is offered as a hypothesis-generating framework that makes explicit how specific combinations of target state, design axes, and formal levers are expected to modulate the viewer’s predictive–affective loop. We outline applications to scene design, film and media education, experimental and observational research, and human–AI collaborative directing, and we indicate how its central quantities can be measured. PAL-D introduces no new psychological principles; its contribution is an integrative, predictive structure rather than a new mechanism. It is a mid-level model specialized for passive viewing, and it treats story-level construction, production conditions, and individual and cultural differences as wider-scope factors, isolating the segment-level predictive–affective trajectory through which these factors act. The framework thus aims to provide a common predictive–affective vocabulary through which filmmakers, media psychologists, and cognitive scientists can jointly analyze and test director-side design of visual narratives.

## 1 Introduction

Audiovisual directing is often described as “the art of expressing emotion,” yet directors still lack a precise way to describe how specific formal decisions shape the viewer’s temporal trajectory of prediction, arousal, and affect. It is widely assumed that shot size and camera movement, framing and composition, color and brightness, editing rhythm, sound, and music influence emotion and immersion, but this assumption rarely takes the form of a mid-level language that connects concrete formal choices to the viewer’s predictive–affective loop. In particular, there is still no sufficiently formalized cognitive–affective model that lets directors talk, in a systematic way, about how changing one aspect of a scene will modulate prediction error, physiological arousal, the prevailing affective condition, and the time course of the integrated affective condition.

Predictive processing treats the brain as a hierarchical generative model that continuously minimizes prediction error—the discrepancy between internally generated predictions and incoming sensory signals (Clark, 2013; Friston, 2010). In PAL-D, ΔP(t) denotes the moment-to-moment change in the level of prediction error over time. Building on this architecture, PAL-D treats changes in physiological arousal as ΔI without committing to a specific account of how affective experience is formed; on predictive accounts, bodily states are themselves regulated through inference, so that prediction error, bodily state, and the allocation of attention are reflected in ΔI (Seth and Friston, 2016). Different patterns of accumulation and dissipation of ΔI are associated with different affective conditions T and integrated affective conditions E. Event segmentation research further shows that, when people watch continuous action or scene sequences, prediction error spikes at particular “event boundaries,” model updating and memory encoding concentrate at those points, and these boundaries structure the temporal organization of viewing (Zacks et al., 2007; Zacks, 2020). However, this line of work remains almost entirely on the viewer side and has not been organized as a design language that directly links variables such as ΔP, ΔI, AO, T, and E to the formal elements that directors can shape.

At the other end of the gap, film theory and aesthetics, perceptual psychology, and phenomenological and embodied film studies have developed rich vocabularies for describing which formal choices are associated with which kinds of felt experience. This tradition carefully analyzes how framing and composition, contrast in brightness and color, shot size and camera movement, editing rhythm, sound, and music shape perception and affect, empathy and immersion. Yet these accounts are usually organized in aesthetic or narrative coordinates rather than in predictive–affective ones. As a result, there is a conceptual disconnect between cognitive neuroscience and film/directing theory: both are well developed on their own terms, but differences in language and coordinate systems make it difficult to connect their results in a way that can guide concrete scene design. Throughout, scene (and scene design) refers to a finished, edited segment as it appears in the final cut, not a single recorded shot or take.

In the present article, we define the viewer-side component as a Predictive–Affective Loop (PAL) model, drawing on predictive processing, research on emotion and appraisal, event segmentation research, and film and directing theory. Section 2 defines only the elements of this viewer-side component that are needed for PAL-D, so that the article can be read as a self-contained Hypothesis and Theory proposal. PAL treats “affectively loaded cognitive episodes” in passive viewing of audiovisual content as structured by a four-layer architecture—Gaze/Input (G), Prediction (P), Appraisal (A), and Integration (E)—and by the interaction of five time-varying quantities ΔP(t), ΔI(t), AO(t), T(t), and E(t). Changes in prediction error ΔP are treated not as the content of emotion but as the primary signal that initiates model updating within the loop and modulates the subsequent organization of ΔI, AO, T, and E. ΔP and ΔI are modeled as partially independent processes that co-vary through shared causes and interactions between bottom-up and top-down processing; AO denotes a set of multiple appraisal outputs produced at the Appraisal layer; T denotes the dominant affective condition prevailing at a given moment, not a discrete emotion category; and E denotes the integrated affective condition at the episode level, in which the prevailing T is bound to memory, self-related processing, and social context. Through these layers and variables, PAL aims to capture viewing as a sequence of recurrent predictive–affective loops.

By design, PAL is viewer-side: it is well suited to describing how viewers allocate gaze (G), form and revise predictions and manage prediction errors (P, ΔP), evaluate errors and cues to generate multiple appraisal outputs AO (A), and converge toward a dominant affective condition T and an integrated affective condition E (E). On its own, however, PAL does not specify what target states a director should aim for, or which design axes and formal choices can be used to reach those states. It does not answer, in director-side terms, which ΔP–ΔI–AO–T–E trajectory should be designed for a given scene. A cognitive–affective grid for viewers is in place, but there is still no corresponding director-side grid that lets directors use this structure as a practical language for scene design.

The PAL-D (Predictive–Affective Loop for Directors) model proposed in this Hypothesis and Theory article addresses this gap by adding a thin design layer on top of the viewer-side PAL model. PAL-D presupposes the same four layers G, P, A, and E and the same five variables ΔP, ΔI, AO, T, and E, but defines the director’s target predictive–affective configuration at the level of scenes as a target state TS. It then decomposes TS into four higher-level design axes—Sensory Density (S), Framing and Focus (F), Narrative–Contextual Cue (Ac), and Affective–Empathic Cue (Ec)—and six perceptual–formal levers that together serve as a design language. In PAL-D, the director’s target is not an everyday emotion label such as “moving” or “suspenseful,” but a desired trajectory of how ΔP(t), ΔI(t), AO(t), T(t), and E(t) should evolve over time. ΔP(t) is treated as the primary organizing dimension of the predictive–affective structure, while ΔI(t), AO(t), T(t), and E(t) are understood as auxiliary dimensions that typically covary with the pattern of ΔP. PAL-D proposes four representative scene-level target-state patterns—calm (TS_calm), tension (TS_tension), crisis (TS_crisis), and relief (TS_relief)—as ΔP-centered predictive–affective patterns whose hypothesized ΔP–ΔI–T trajectories and proposed S/F/Ac/Ec profiles are later summarized in a table (Table 1).

**Table 1:** Four proposed representative target states (TS_calm, TS_tension, TS_crisis, TS_relief) and their hypothesized ΔP–ΔI–T trajectories and proposed representative S/F/Ac/Ec profiles in the PAL-D model.

Target state	ΔP(t) trajectory	ΔI(t) and T(t) response	Proposed representative S/F/Ac/Ec profile
TS_calm	ΔP stays low with only small, quickly absorbed fluctuations.	ΔI varies minimally around baseline; T remains calm or neutral over time.	S low to medium; F medium to high and relatively stable; Ac high and coherent, providing clear temporal, spatial, and causal structure; Ec low to medium, with background-level empathy.
TS_tension	ΔP gradually rises from a low level to a medium–high plateau and remains elevated until just before the local climax.	ΔI slowly increases with a slight lag behind ΔP; T shifts from neutral toward an “about to explode” tension condition.	S medium to high; F progressively narrowed onto key characters and cues; Ac medium, with foreshadowing and partial information but maintained ambiguity; Ec medium to high and gradually strengthening as viewers align with focal characters.
TS_crisis	ΔP exhibits a steep spike over a short interval and then remains high or oscillates around a high level.	ΔI shows a sharp peak and sustained high arousal; T is pulled into a threat- or loss-oriented affective condition before any new model is stabilized.	S high or abruptly reconfigured; F very high, concentrating attention on a single locus or small region; Ac initially low, with unexplained threat and causal gaps, then quickly increasing as the threat becomes explicit; Ec high, with strong identification and concern for the characters.
TS_relief	ΔP starts from a high state (often following TS_crisis) and rapidly falls to a low, stable range.	ΔI decreases more slowly than ΔP, showing a delayed return toward baseline; T shifts from tension to a relief-oriented affective condition and then stabilizes.	S medium in a re-stabilized environment; F medium, including both characters and contextual information; Ac high and integrative as prior cues are woven into a coherent narrative; Ec medium to high as viewers track characters’ emotional adjustment and closure.

BOX 1 **Key viewer-side quantities and director-side axes in PAL and PAL-D.**

- *ΔP(t):* change in prediction error; not the content of emotion but the primary organizing signal that initiates and modulates model updating within the loop.
- *ΔI(t):* change in physiological arousal (I = intensity), reflecting prediction error, bodily state, and allocation of attention; modulated by ΔP but partly independent.
- *AO(t)* (appraisal output): a distribution over multiple co-existing interpretive hypotheses (AO₁, AO₂, …); multivalued.
- *T(t):* the dominant affective condition prevailing at a given moment (not a discrete emotion category).
- *E(t):* the integrated affective condition at the episode level (specific emotion categorization lies beyond PAL-D’s scope).
- *Layers:* Gaze/Input (G), Prediction (P), Appraisal (A), Integration (E). *Director-side axes:* sensory density (S), framing and focus (F), narrative–contextual cueing (Ac), affective–empathic cueing (Ec). *TS:* target state.

All five viewer-side quantities are time-varying functions of *t*.

The four design axes allow directors to decompose a target TS into an S/F/Ac/Ec profile and to treat “how complex and volatile the environment is (S), where and how strongly gaze is anchored (F), which rules, goals, and relational cues are provided (Ac), and which affective and empathic directions are amplified (Ec)” as key design variables. These axes are implemented through six perceptual–formal levers—camera position and distance, camera movement, framing and composition, light, color, and brightness, editing rhythm and transitions, and sound and music—which modulate the four PAL layers in different ways and thereby tune the ΔP–ΔI–AO–T–E loop. Low S, stable and predictable framing, clear narrative and contextual cues, and moderate affective and empathic cues tend to keep ΔP and ΔI at low overall levels, forming a configuration close to TS_calm. Discontinuous camera movement and abrupt cuts, ambiguous temporal and spatial cues, and unexplained narrative gaps jointly modulate S and Ac to increase the number of competing ΔP candidates, supporting high ΔP–ΔI patterns typically associated with TS_tension or TS_crisis. Close-ups and subjective shots, facial expressions and sound design that track breathing or heartbeat, and empathic music primarily raise Ec and can steepen the gradients of ΔI and T even for the same ΔP. PAL-D thus makes explicit a conceptual chain running from formal choices, through six levers and the S/F/Ac/Ec profile, to the four PAL layers and the ΔP–ΔI–AO–T–E loop, and finally to viewing experience, in order to place directorial intuition on a predictive–affective grid. This grid is meant to supplement the natural language of practice—“tension,” “rhythm,” “letting a moment breathe”—not to replace it. Directors continue to work in their own terms; PAL-D’s role is to make explicit what those terms are shaping, primarily as a layer for analysis, planning, and diagnosis rather than for moment-to-moment speech on set.

**Figure 1:**
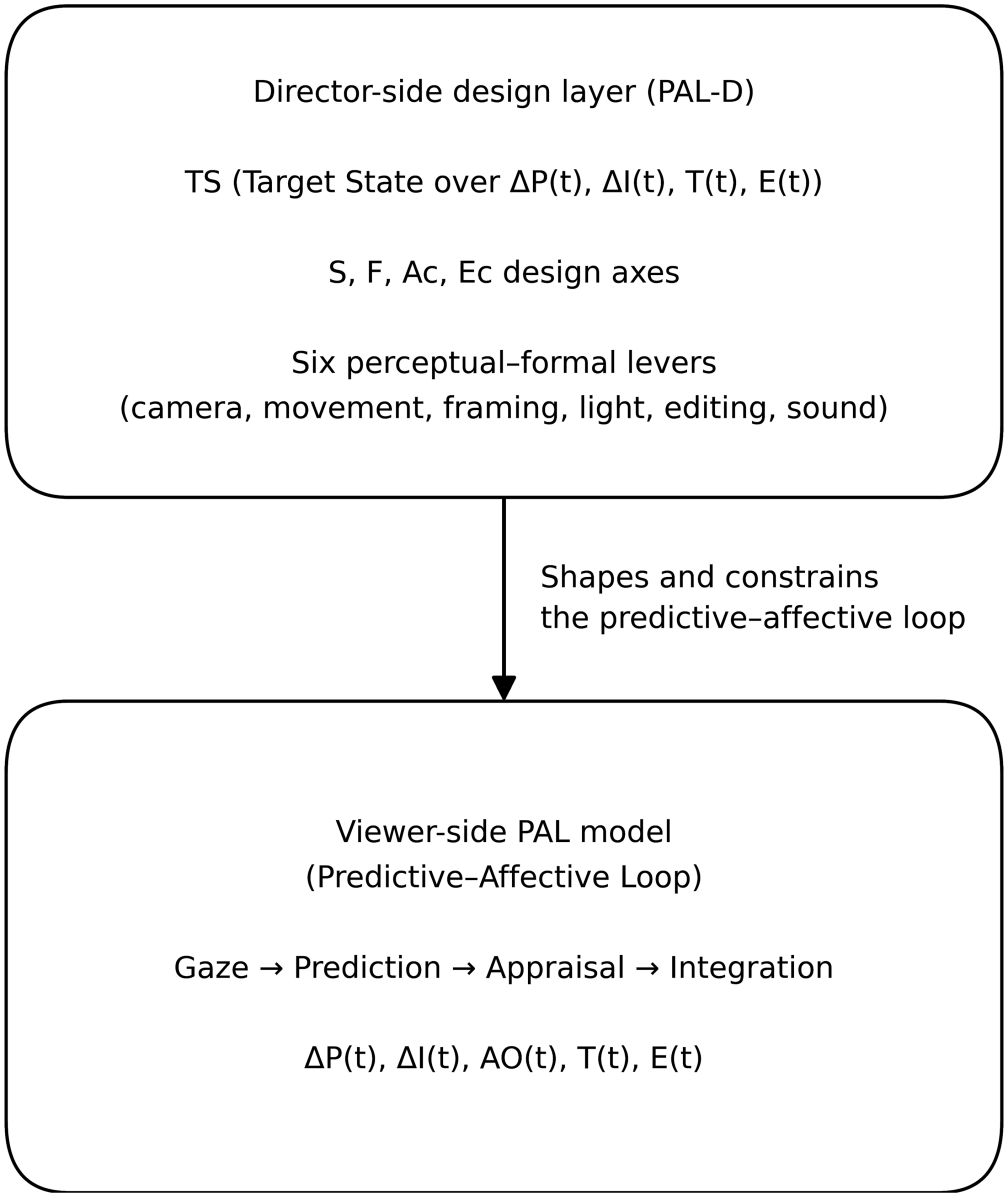
Schematic overview of the director-side PAL-D design layer and the viewer-side PAL model. The director-side PAL-D design layer is shown at the top, specifying a target state (TS), the four design axes (S, F, Ac, Ec), and six perceptual–formal levers (camera position/distance, camera movement, framing/composition, light/color/brightness, editing rhythm/transitions, sound/music/atmosphere). The viewer-side PAL model is shown below as a four-layer predictive–affective architecture (Gaze/Input, Prediction, Appraisal, Integration) in which ΔP(t), ΔI(t), AO(t), T(t), and E(t) are recursively updated over time. The downward arrow indicates that director-side design choices, realized through the levers, shape and constrain the viewer-side predictive–affective loop.

This article does not present empirical data or directly test the PAL-D model. Rather, it offers a conceptual proposal that formalizes a director-side predictive–affective grid by integrating predictive processing, research on emotion and appraisal, event segmentation research, existing film and directing theory, and the viewer-side PAL model, and uses this grid to outline a research agenda for future experimental work, textual analysis, and applications in directing education and practice. PAL-D is not advanced as a general theory of human cognition; it is a mid-level model specialized for situations in which viewers passively watch audiovisual content. Story structure and large-scale construction in long-form narratives, production conditions and industrial structures, individual and cultural differences, and platform contexts are deliberately kept outside its scope. These factors are bracketed not because they are irrelevant but to isolate a single level of analysis—the segment-level predictive–affective trajectory through which they ultimately act. Story content and cultural or individual priors, for example, shape the predictions and prior expectations that enter this trajectory rather than bypassing it; PAL-D fixes the trajectory as its object and treats these larger factors as inputs that wider-scope work can later connect to it. The aim is to bring the director’s intuitive language and the viewer’s predictive–affective architecture into a shared coordinate system. In doing so, PAL-D introduces no new psychological principles: its constructs—prediction error, physiological arousal, appraisal, affective conditions, and event segmentation—are drawn from existing research. Its contribution is an organizing, predictive structure that places director-side design decisions and the viewer’s predictive–affective dynamics within a common, time-resolved coordinate system—a way of connecting and jointly testing existing findings rather than positing a new mechanism. The remainder of the article proceeds as follows. Section 2 offers a compact theoretical background and formally defines the viewer-side PAL model. Section 3 lays the PAL-D design grid on top of PAL, defining TS, S/F/Ac/Ec, and the six levers and summarizing the overall architecture through Figure 1 and Table 1. Section 4 shows how the predictive–affective design language proposed by PAL-D can be used in scene analysis, directing education, and the design of empirical studies. Section 5 discusses the theoretical implications of PAL-D as a bridge between audiovisual directing studies, cognitive neuroscience, emotion research, and media practice. Section 6 then addresses limitations, viewer divergence, and future directions, including the boundary conditions under which intended target states may diverge from observed viewer trajectories.

## 2 Theoretical background: predictive–affective viewing and the viewer-side PAL

To introduce PAL-D, we first adopt a perspective that treats viewing as a predictive–affective process and, on this basis, formally specify the four layers and five variables of the viewer-side PAL model. PAL-D adds a thin director-side design grid on top of PAL, which already describes the viewer’s internal predictive–affective loop. The basic structure of PAL and the role of each of its elements therefore have to be defined with sufficient precision; otherwise the design language of PAL-D will lack stability. In this section, we briefly review key insights from predictive processing, research on emotion and appraisal, event segmentation research, and existing film theory, and then formalize the viewer-side PAL model, which structures viewing as an “affectively loaded cognitive episode” in terms of a predictive–affective loop with four layers and five time-varying quantities ΔP(t), ΔI(t), AO(t), T(t), and E(t).

Predictive processing theory understands the brain not as a system that passively receives stimuli, but as a system that constantly predicts future input via a hierarchical generative model and attempts to minimize the difference between this prediction and actual input (Clark, 2013; Friston, 2010). In this view, the brain is a prediction machine that responds sensitively to the discrepancy between predictions generated by the internal model and incoming sensory signals, and changes in this discrepancy are captured by prediction-error change ΔP. ΔP is not a static measure of error size; it functions as a dynamic signal that indicates how stable the current model is, whether new hypotheses are needed, and whether behavior should be adjusted. In PAL and PAL-D, ΔP is not treated as the content of emotion itself but as a primary signal that initiates and modulates model updating within the predictive–affective loop. The starting point of PAL is that how ΔP arises within a scene, how long it is maintained, and at what point and with what speed it decays will, in practice, shape the subsequent temporal organization of ΔI, AO, T, and E.

Predictive accounts of affect link this architecture to bodily states and felt experience through interoceptive inference (Seth and Friston, 2016). The mismatch between predictions about internal bodily states and interoceptive input appears as change in physiological arousal ΔI, where I denotes intensity. In PAL and PAL-D, ΔP is treated as the primary signal that modulates ΔI, and ΔI in turn is treated as a partly independent factor that influences appraisal and affective integration. Crucially, ΔI and AO are not bound together in a single linear chain of cause and effect. AO (appraisal output) is conceptualized as a set of multiple cognitive hypotheses used to interpret prediction error and context, that is, a distribution over several appraisal outputs AO₁, AO₂, …, whereas ΔI denotes changes in physiological arousal that reflect prediction error, bodily state, allocation of attention, and related factors. Both AO and ΔI are partially mediated by ΔP and can interact, but PAL and PAL-D do not reduce their relationship to the narrative that appraisal generates arousal and arousal generates emotion (Scherer, 2005, 2009; Seth and Friston, 2016). The basic assumption of PAL and PAL-D is that the affective condition unfolds over time through the interaction of ΔP, ΔI, AO, T, and E. Within this loop, ΔP functions as the signal that initiates and tunes model updating within the loop, ΔI represents changes in physiological arousal, AO denotes the distribution of interpretive hypotheses, and T and E denote, respectively, the dominant affective condition and the integrated affective condition that emerge from their interaction.

At the level of the Integration layer, AO and ΔI, together with past experience and social norms, tend to converge into a dominant affective condition T and an integrated affective condition E at particular moments. PAL and PAL-D treat AO as a multivalued structure, that is, as a set of multiple interpretive hypotheses AO₁, AO₂, … that coexist and compete over time. By contrast, T (the dominant affective condition) is the affective condition prevailing at a given moment: among the affective possibilities present, one becomes dominant and constitutes the prevailing condition. Viewers can entertain several interpretations of a scene at once—for example, that a character may be in danger or that the situation may be a misunderstanding—but their affect at that moment is often experienced as a single dominant condition, such as diffuse anxiety or heavy sadness. E (the integrated affective condition) denotes the integrated affective condition at the episode level, in which this T is bound to memory, self-representation, and social norms. Assuming a multivalued structure for AO and a prevailing dominant condition for T, PAL holds that changes in the distribution of AO and the interaction among ΔP, ΔI, AO, T, and E together mediate the formation of T and E. In this article, the viewer-side PAL model is therefore described as a ΔP–ΔI–AO–T–E loop. However, in the later definition of PAL-D and in the discussion of TS, S/F/Ac/Ec, and the levers, we focus on the temporal patterns of ΔP, ΔI, T, and E and treat AO as a latent variable in order to reduce notational and explanatory complexity. In other words, even when AO is not explicitly written as a symbol in the PAL-D exposition, its multivalued structure and mediating role within the ΔP–ΔI–AO–T–E loop remain part of the conceptual background.

With respect to ΔI, PAL and PAL-D explicitly assume that ΔI tends to follow the pattern of ΔP with a characteristic temporal lag. The actual delay of physiological responses can vary from several hundred milliseconds to several seconds depending on stimulus type, context, and individual differences, and this article does not commit to specific numerical values. Instead, we adopt the more general assumption that there are typical trajectories in which ΔI rises or falls after a sharp change in ΔP with some delay. ΔI is not a single index but a multidimensional construct that can be operationalized as a combination of physiological measures such as skin conductance, heart rate, heart-rate variability, and respiration. Designing how ΔI should be estimated and combined in empirical work remains an independent methodological task for future studies. In this article, PAL and PAL-D therefore use ΔI as a mid-level variable that can later be instantiated on top of more detailed measurement strategies.

Event segmentation research provides empirical evidence about how this predictive–affective architecture is organized over time. When people view continuous actions or sequences of scenes, they spontaneously detect event boundaries, and at these points prediction-error change ΔP tends to increase temporarily; model updating and memory encoding also tend to concentrate there (Zacks et al., 2007; Zacks, 2020). Empirical work on continuity editing supports this graded relationship: discontinuities in action have a substantially larger effect on viewers’ event segmentation than discontinuities in space or time, and commercial editing is shaped to support the comprehension of events across breaks in low-level visual continuity (Magliano and Zacks, 2011). From the perspective of PAL and PAL-D, event boundaries are therefore closely linked to points at which ΔP rises sharply. However, this does not justify the simple equation “high ΔP equals event boundary.” Some spikes in ΔP are resolved at the level of micro-surprise and do not stabilize as meaningful narrative event boundaries, whereas some relatively large structural transitions are anticipated by viewers and pass smoothly without steep surges in ΔP. What distinguishes the two cases is whether a manipulation violates the viewer’s current event-model predictions—the condition under which event boundaries are perceived in the first place (Zacks et al., 2007). Because ΔP is by definition the change in prediction error, editing or boundary placement counts as ΔP modulation specifically when a cut, transition, or restructuring forces the current model to be revised. When the same devices instead redistribute attention, reorganize narrative information, or support memory encoding without violating those predictions, they act on attention and event structure but not, in themselves, on ΔP. In this article, PAL-D treats patterns of ΔP and event boundaries as two levels of structure that largely overlap but are not identical. When directors decide where to amplify prediction error and where to resolve it, and which points to turn into event boundaries, the patterns of ΔP–ΔI–AO–T–E become the key criteria, and director-side PAL-D treats these decisions as central design targets.

Film theory and directing theory, for their part, have long provided a rich vocabulary at the other end of this gap. Arnheim and the Gestalt tradition analyzed how framing and composition, balance and asymmetry, and contrasts of brightness and color shape a perceptual whole, exploring how the organization of the frame structures perception itself (Arnheim, 1974). Münsterberg and early psychological film theory examined how film organizes and coordinates mental functions such as perception, attention, and memory, and discussed how close-ups and montage can restructure psychological focus and the flow of thought (Münsterberg, 1916). Later cognitive film theory analyzed how narration cues and constrains viewer inference (Bordwell, 1985), how formal elements such as shot size and camera movement, point-of-view and subjective shots, editing rhythm and transitions, and sound and music influence attention, empathy, and affective engagement (Tan, 1996), and how suspense arises when a narrative question poses two opposed outcomes of unequal likelihood (Carroll, 1984); phenomenological and embodied approaches described the lived, embodied structure of the film experience itself (Sobchack, 1992). These bodies of theory are powerful in explaining how particular formal choices allow directors to create scenes that feel a certain way, but they are largely organized in aesthetic and narrative coordinate systems and are not yet formulated in a coordinate system that is directly compatible with variables such as ΔP, ΔI, AO, T, and E used in predictive processing and research on emotion and appraisal. PAL and PAL-D aim to provide a mid-level common grid at this junction that can connect cognitive neuroscience with film and directing theory.

Against this background, the viewer-side PAL (Predictive–Affective Loop) model is specified here as a mid-level cognitive–affective framework. PAL structures affectively loaded cognitive episodes that occur during passive viewing of audiovisual content by means of a hierarchical architecture of four processing layers—Gaze/Input (G), Prediction (P), Appraisal (A), and Integration (E)—and the interaction of the five time-varying quantities ΔP(t), ΔI(t), AO(t), T(t), and E(t). In doing so, it offers a predictive–affective account of what happens inside the viewer. The Gaze/Input layer encompasses both the physical characteristics of the input and early perceptual processing, and the selection of gaze and attention. PAL and PAL-D focus on gaze and attention as the primary level at which director-side choices can indirectly influence signals that can, in principle, be measured, and in practice treat the G layer as a sampling layer that determines which information is sampled, where, and when. The fourth layer is labeled Integration (E) because it denotes the level at which the episode-level outcome E(t) is integrated, that is, the affective condition at the level of the episode. To avoid confusion, we consistently distinguish in the text between the Integration layer (E layer) and the affective condition E(t) when we refer to layer names and variable names. The term “loop” denotes not a one-way chain that runs once from input to prediction, error, arousal, and emotion, but a recurrent structure in which outputs at each layer feed back to other layers, such that prediction and error, arousal and appraisal, and integration are recursively recalibrated over time.

The roles of the four layers can be summarized in functional terms. At the Gaze/Input layer, it is determined which locations and objects within the frame and across the screen are selected and enter as input. Viewers do not process all available information at once; they sample a subset of elements through gaze and attention. Visually salient features such as strong contrast, motion, or faces attract gaze in a bottom-up manner, while narrative importance, genre expectations, and predictions formed in previous scenes guide where to look in a top-down fashion. From the perspective of PAL, the G layer defines the input set that will serve as the basis of all subsequent predictions and appraisals. At the Prediction layer, the internal generative model of the current scene state and the near future is formed and updated based on the sampled input. This layer organizes hypotheses about what is happening in the scene and what is likely to happen next. The discrepancy between these hypotheses and the actual input appears as prediction-error change ΔP(t). When ΔP is small, it signals that the current model explains the environment sufficiently; when ΔP is large, it signals that the model needs to be revised or that new hypotheses are required.

At the Appraisal layer, prediction, actual input, and contextual information are evaluated in terms of their meaning and significance (Scherer, 2005, 2009; Scherer and Moors, 2019). Where appraisal theory is invoked, it marks correspondences between processing states and appraisal dimensions (novelty, certainty, control), not a claim that appraisal causally generates emotion; on this view appraisal differentiates emotional components over time and categorical labeling is a later step (Scherer and Moors, 2019). AO(t) is conceptualized here as the temporal distribution of multiple appraisal hypotheses AO₁, AO₂, … about what has happened, why it happened, and what implications it may have. Each AO leads to a different interpretation of the magnitude and direction of ΔP and can therefore alter future predictive strategies and readiness for action. PAL and PAL-D emphasize that the distribution of AO is constrained by ΔP and at the same time feeds back into the future course of ΔP. ΔP modifies the distribution of AO, and AO, by changing how serious this error is judged to be and what consequences it is expected to have, indirectly constrains the subsequent trajectory of ΔP. Again, AO and ΔI interact via ΔP; they are not modeled as a simple chain in which one directly generates the other.

At the Integration layer, prediction-error change ΔP, changes in physiological arousal ΔI, the affective–cognitive interpretations represented by AO, and higher-order cognitive factors such as memory, norms, and self-representation are integrated into T(t) and E(t). T(t) is the prevailing affective condition at a given moment—the overall tone of a scene—rather than a discrete emotion category. E(t) is the integrated affective condition at the episode level. Which specific nameable emotion a viewer reports—such as fear, anger, or being moved—depends on genre, alignment, culture, and individual differences, and lies beyond PAL-D’s scope, which describes the design and change of affective conditions and refers specific emotional experience to existing emotion research, consistent with evidence that the affective response to uncertainty is moderated by context and individual differences rather than fixed (Anderson et al., 2019). PAL treats ΔP, ΔI, AO, T, and E not as a static set of variables but as components of a recursive loop in which prediction and error, arousal and appraisal, and integration repeatedly interact. A single affectively loaded cognitive episode is defined here as one pass of this predictive–affective loop. Put differently, PAL describes watching a scene as repeatedly sampling what is on screen, guessing what will happen next, evaluating what it means, and folding these evaluations into a felt mood and a more specific affective episode over time.

In short, the viewer-side PAL model provides a basic grid of four layers and five time-varying quantities for describing viewing in predictive–affective terms. By distinguishing ΔP as a primary signal that initiates and modulates model updating within the predictive–affective loop rather than as the origin or content of emotion, ΔI as change in physiological arousal, AO as a distribution of multiple interpretive hypotheses, T as the dominant affective condition, and E as the integrated affective condition, PAL brings together predictive processing, research on emotion and appraisal, event segmentation research, and film and directing theory within a single temporal structure. PAL-D takes this viewer-side grid as its premise and then defines director-side design variables on top of it. The conceptual relationship between the four PAL layers, the five time-varying functions, and the thin PAL-D design layer is summarized schematically in Figure 1, and the next section develops the PAL-D model by extending this viewer-side grid into director-side target states (TS), S/F/Ac/Ec, and six levers.

## 3 PAL-D model: target states, design axes, and perceptual–formal levers

In this section, we define the core components of the PAL-D model—target states (TS), four higher-level design axes (S, F, Ac, Ec), and six perceptual–formal levers—and spell out how they modulate the four viewer-side PAL layers and the ΔP–ΔI–AO–T–E structure. Because PAL-D adds a director-side design grid on top of PAL, every element has to correspond directly to the viewer’s internal cognitive–affective processes while at the same time functioning as a design language that directors can use. The concepts of PAL-D are therefore required to do double work: they must describe what happens inside the viewer and specify which design changes are expected to modulate the viewer-side trajectory. TS is defined here as an intended predictive–affective trajectory that the director assumes at the design stage and is treated as a baseline that can later be compared to actually observed ΔP–ΔI–T–E patterns in empirical work.

In PAL-D, a target state (TS) is the temporal pattern of predictive–affective structure that the director aims to achieve within a particular scene or continuous segment. Rather than everyday emotion labels such as “being moved” or “feeling tense,” a TS specifies how ΔP(t), ΔI(t), AO(t), T(t), and E(t) are intended to evolve over time. Formally, one could imagine specifying a target pattern for all five functions, but PAL-D assumes that AO (appraisal output) is already present in PAL as a multivalued intermediate structure and therefore characterizes TS primarily in terms of the temporal structure of ΔP, ΔI, T, and E, treating AO as a latent variable whose effects are projected into these patterns. Within this framework, ΔP(t) is treated as the primary organizing axis, and ΔI(t), T(t), and E(t) are understood as secondary axes that typically align with the pattern of ΔP. A director’s intention to create “slowly mounting tension,” for example, can be described as a trajectory in which ΔP is low in the early part of the scene, gradually rises in the middle, peaks near the climax, and then either remains at a plateau or converges slowly. ΔI then follows with a modest temporal lag, and T shifts from a near-neutral condition toward an anxious or tense condition. For this reason, AO is not written explicitly at every step in the PAL-D exposition, even though the full ΔP–ΔI–AO–T–E loop is assumed as the conceptual background. A TS is the director’s intended, ΔP-centered predictive–affective trajectory for a segment. The four representative types—calm, tension, crisis, and relief—are classes of such intended trajectories, not separate definitions. The pattern actually realized in viewers is an observed trajectory, distinct from the TS and serving as independent evidence for how closely a TS was achieved. Because a TS is defined over a finished segment, it is neutral with respect to production stage: the same intended trajectory may be realized through shooting, through editing, or through both. A TS is also an analytic and pedagogical construct, characterizing the intended trajectory of a segment whether that intention is set explicitly in advance or reconstructed afterward from loosely planned, emergent, or collaborative work. The degree to which a director sets such targets explicitly is itself a variable the framework can describe, not a precondition for applying it. PAL-D is therefore intended less as a real-time tool on set than as an analytic and design language for pre-production—planning a segment’s intended trajectory, including how it will be shot and cut—and post-production—diagnosing how the edited segment realizes or departs from that trajectory. Its unit of analysis is the finished, edited segment and the intended trajectory inscribed in it, not the authorship behind it; whether that trajectory issues from a single author or from a distributed, collaborative process lies outside the framework’s claims and does not affect its application.

This article proposes four representative TS patterns in PAL-D, organized around the temporal structure of ΔP, referred to as a calm TS (TS_calm), a tension TS (TS_tension), a crisis TS (TS_crisis), and a relief TS (TS_relief). In TS_calm, ΔP remains in a low range with only small fluctuations, ΔI is kept at low intensity, and T shows minor ripples around a calm or neutral condition. In TS_tension, ΔP follows a gently rising trajectory over a certain interval, ΔI responds with a slight delay and climbs slowly, and T converges toward a condition of “about to explode” tension. In TS_crisis, ΔP shows a sharp spike over a short period, ΔI reacts strongly immediately afterward, and T swings rapidly toward threat- or loss-oriented affective conditions and remains there until a new model is constructed. In TS_relief, ΔP starts at a high level and drops quickly to a lower range within a relatively short time, ΔI decreases more slowly than ΔP, and T shifts from tension toward a relief-oriented affective condition and then converges back toward a stable state. PAL-D proposes these four TS types in terms of their characteristic ΔP–ΔI–T patterns and uses them as working hypotheses when discussing which TS a scene is designed to target and where transitions between TS types are placed within a sequence. These four patterns broadly match prototypical affective flows discussed in narrative structure, tension and suspense, and event segmentation research (Tan, 1996; Lehne and Koelsch, 2015; Zacks et al., 2007), and PAL-D reformulates them on a ΔP-centered predictive–affective coordinate system. The four TS types, together with TS-specific ΔP–ΔI–T patterns and proposed, representative S/F/Ac/Ec profiles, are conceptually summarized in Table 1 as prototypes at the level of group-level averages that can serve as practical references for scene design and empirical study. Because PAL-D does not commit to any particular account of how specific emotion categories arise, and treats T and E as affective conditions rather than fixed emotion categories (Section 2), the four TS types should be read in the same spirit. They are not a closed set of fixed emotional categories. At the level of the ΔP skeleton—the temporal shape of prediction-error change that defines each type—they are relatively general, since this shape draws on broad regularities of prediction and event structure (Zacks et al., 2007). At the level of affective realization—which affective condition a given skeleton is associated with—they vary with genre, culture, and individual viewer. And they are a small set of representative design patterns that can be combined, overlapped, and graded, rather than an exhaustive taxonomy into which every scene must fall. On this reading, the relative stability of the four types and the dynamic, condition-based view of affect in Section 2 are consistent: the stability lies in the ΔP skeleton, while the affective realization remains variable.

Each TS can be further decomposed into four higher-level design axes. PAL-D defines these axes as sensory density (S), framing and focus (F), narrative–contextual cueing (Ac), and affective–empathic cueing (Ec). S denotes the total amount of sensory and informational processing that a scene demands and functions as the axis along which the director decides how densely to pack the environment with input. High S describes frames or sequences with many visual and auditory elements that must be processed at once, with rich detail and numerous potential sources of ΔP and cognitive load. Low S describes restricted information, low perceptual and cognitive load, and ample “empty space,” that is, an environment with relatively few candidates for ΔP and high environmental stability. Even when showing the same diegetic space, a director can create high S by densely arranging many characters and objects and layering complex sound, whereas simplifying the arrangement and presenting minimal information results in low S.

F regulates where gaze is drawn, how strongly it is held, and how concentrated or dispersed the informational field is. It is implemented through composition, camera distance and angle, depth of field, and camera movement, and strongly biases where appraisal hypotheses are likely to form. Where viewers look is shaped by visual salience and scene structure as part of front-end attentional selection, and F denotes the director-side means of biasing this selection (Loschky et al., 2020, 2026). Deep focus, complex mise-en-scène, and a large number of characters and objects within one composition create lower F, with gaze and information more widely dispersed. Shallow focus, strong close-ups, simplified backgrounds, and stable gaze guidance correspond to higher F, concentrating attention on one or a few loci. Under similar S conditions, higher F allows viewers to locate “where to look” relatively quickly, whereas lower F extends the period in which AO is dispersed across multiple competing candidates.

Ac regulates the density and coherence of cues related to rules, goals, relationships, risks and rewards, time and place, and social context. It comprises information that helps viewers build a situational frame for what game is being played, what is at stake, and how characters are related. This situational frame corresponds to the dimensions along which viewers index and integrate events into situation models—time, space, causation, goals, and protagonists and their relations—both in narrative comprehension generally (Zwaan and Radvansky, 1998) and in film specifically (Magliano et al., 2001). In TS_calm, temporal, spatial, and causal relations are clearly specified, yielding high Ac. In TS_tension, foreshadowing cues hint at upcoming events, but some information is deliberately omitted or presented ambiguously, keeping Ac at an intermediate level. In TS_crisis, an initial phase of low Ac—unexplained threats, causal gaps, broken rules—drives sharp increases in ΔP, followed by a phase in which explicit threat or catastrophe cues appear and a new model is constructed. In TS_relief, narrative and contextual cues accumulated in preceding tension and crisis segments are organized into a coherent story, Ac rises, and ΔP converges quickly. Ac is thus modeled as regulating both meaning evaluation at the Appraisal layer and the richness and coherence of priors at the Prediction layer. Low Ac makes it easier for a given input to generate large ΔP, whereas high Ac facilitates rapid model reconstruction and convergence even after large ΔP.

Ec adjusts the quantity and quality of affective and empathic cues related to characters and situations. It is implemented through facial expression and gaze, bodily posture and movement, camera distance and point of view, sound design and music, and the content and tone of dialogue, as well as reaction shots and editing patterns. Treating affective and empathic cues as coordinated across style and narrative to shape the viewer’s affective orientation over time, rather than to dictate a discrete emotion, draws on the mood-cue account of filmic emotion (Smith G. M., 2003); the way such cues structure the viewer’s engagement with characters—through alignment and allegiance—draws on the structure of sympathy (Smith M., 1995). In PAL-D, Ec is modeled as a major influence on ΔI, T, and E. Given the same ΔP pattern, higher Ec is expected to amplify ΔI and steepen the gradient of T. At the same time, Ec alters evaluations of how important an event is for the viewer, thereby indirectly regulating which candidate models are strengthened at the Prediction layer and how much ΔP is allowed to persist. The same narrative event may be followed by relatively quick convergence of ΔP when empathy is low, whereas high Ec can sustain the same level of ΔP for longer and be associated with stronger affective conditions. Ac is therefore modeled as a primary regulator of priors and ΔP patterns, while Ec is modeled as a primary regulator of the gradient and intensity of ΔI, T, and E, with the two axes modulating the ΔP–ΔI–T–E loop in different ways at the Prediction, Appraisal, and Integration layers. In practice, narrative information and empathic cues are often implemented together within the same shots and scenes, so Ac and Ec frequently covary. PAL-D analytically separates these two axes so that future empirical work can estimate their respective contributions to ΔP and to ΔI, T, and E. Put differently, S corresponds roughly to how crowded the frame feels, F to how clearly the scene tells you where to look, Ac to how clearly you understand what game is being played, and Ec to how much you are invited to care about the characters.

The axes S, F, Ac, and Ec can be used with the same notation when describing viewer states within PAL and when defining director-designed TS in PAL-D. Unless otherwise specified, PAL-D interprets S, F, Ac, and Ec as director-side design axes and reserves superscripts such as Sᵛ and Fᵛ for explicit reference to viewer-side states. Conceptually, the four axes are proposed as analytical dimensions rather than established independent factors, and combinations such as high S with low F or high Ac with low Ec are all permitted within the PAL-D design space. In actual scenes, however, S and F, F and Ec, and Ac and Ec will often move together—for example, a close-up simultaneously concentrates gaze (raising F) and foregrounds a character’s facial expression (raising Ec)—and within certain scene types or genres particular correlation patterns among S, F, Ac, and Ec may emerge. PAL-D treats such patterns as properties of real-world implementations and notes the need for empirical studies, for example using factor analysis, to examine how the correlation structure among the four axes varies across genres, directorial styles, and cultures.

Directors do not implement TS and S/F/Ac/Ec only as abstract goals; they realize them through six perceptual–formal levers. In PAL-D, these levers are grouped as camera position and distance, camera movement, framing and composition, light, color, and brightness, editing rhythm and transitions, and the sound environment. Camera position and distance regulate psychological distance and information density, and camera movement shapes perceived energy and stability and the trajectory of gaze. Framing and composition determine which elements are grouped into a Gestalt within the frame and what is perceived as central. Light, color, and brightness shape visual contrast, atmosphere, and the sense of time and space. Editing rhythm and transitions regulate where, when, and how strongly ΔP spikes by controlling shot duration, regularity, and the placement and type of cuts and transitions, which tend to coincide with event boundaries without being identical to them. The sound environment, through music, sound effects, ambience, and silence, can strongly modulate ΔI, T, and E. Practically, high S can be created by combining complex mise-en-scène, short shot durations, and dense sound; high F by close-ups and shallow depth of field, centralized composition, and stable camera work; high Ac by expository shots, clarifying dialogue, and causal editing; and high Ec by facial close-ups, point-of-view shots, emotive music, and reaction shots.

The six levers affect the four PAL layers in different ways. Camera position and distance, framing and composition, and light, color, and brightness primarily act on the Gaze/Input layer by determining what is perceived as a unit and what is seen first. Editing rhythm and camera movement regulate gaze shifts and the placement of cuts and transitions at the Gaze and Prediction layers; because these tend to coincide with event boundaries without being identical to them, they shape rather than determine the magnitude and timing of ΔP. Narrative–contextual cueing is mainly implemented through editing, framing, dialogue, and sound design that contribute to Ac, shaping the formation and revision of the AO distribution at the Appraisal layer and influencing how priors are filled and updated at the Prediction layer. Elements that contribute strongly to Ec—such as character close-ups and subjective shots, sound design that tracks breathing or heartbeat, emotive music, and reaction shots—can strongly modulate ΔI, T, and E, change evaluations of how important an event is, and thereby indirectly influence model selection and updating strategies in the predictive system. PAL-D’s aim is to make this chain explicit: from formal choices to six levers, from levers to S/F/Ac/Ec, from S/F/Ac/Ec to the four PAL layers, from the layers to the ΔP–ΔI–AO–T–E loop, and finally to viewing experience.

**Figure 2:**
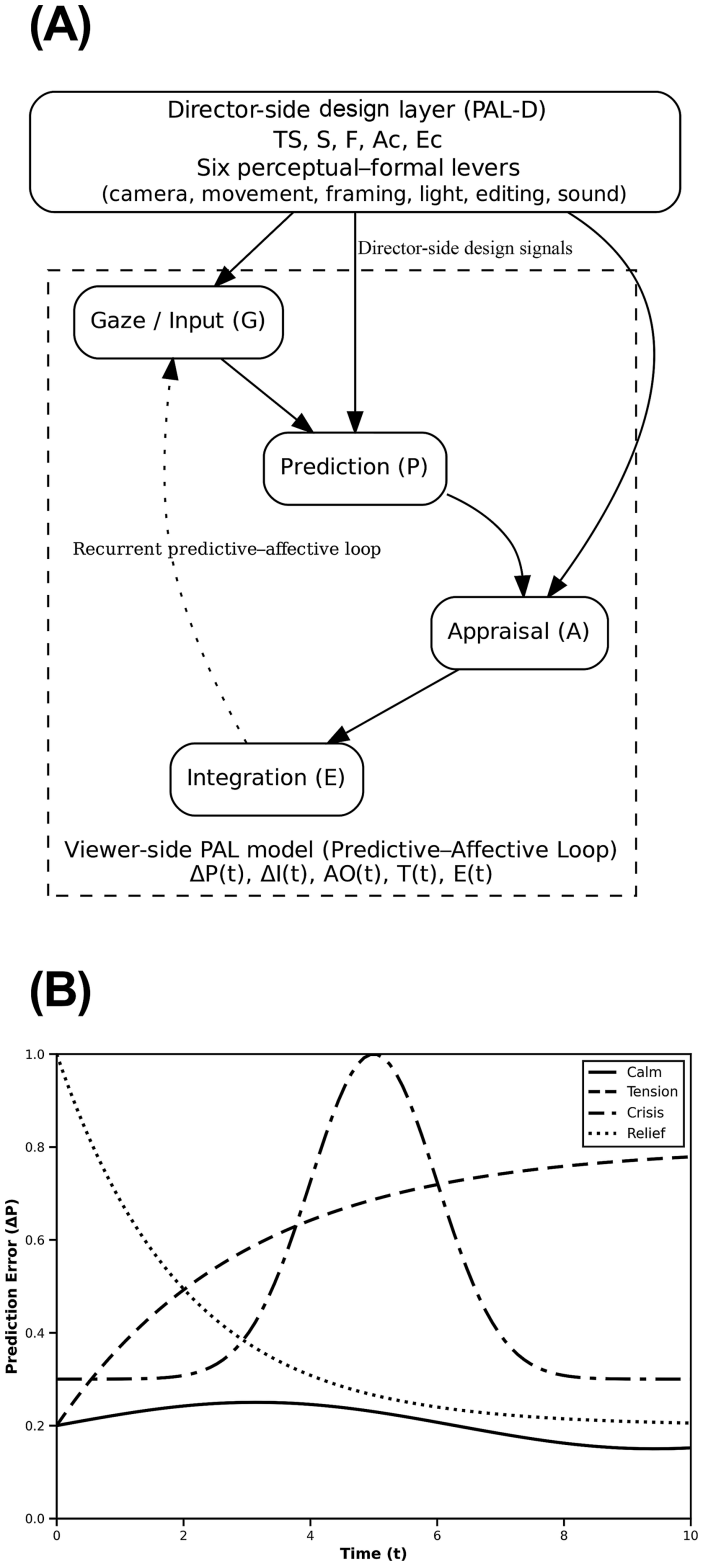
Director-side design structure and ΔP-centered target-state patterns in the PAL-D framework. **(A)** Illustrates the director-side design layer, in which a selected target state (TS_calm, TS_tension, TS_crisis, or TS_relief) is coupled with a specific S/F/Ac/Ec profile and implemented through six perceptual–formal levers. The resulting design signals are modeled as biasing the four PAL layers (Gaze/Input, Prediction, Appraisal, Integration), modulating the computation of ΔP(t), ΔI(t), AO(t), T(t), and E(t) over time. **(B)** Shows prototypical ΔP(t) trajectories for the four target states, emphasizing that PAL-D defines TS not as a verbal emotion label but as a characteristic ΔP-centered temporal pattern (low and stable for TS_calm, gradually rising and sustained for TS_tension, sharply spiking for TS_crisis, and rapidly decaying from a high initial level for TS_relief). These curves are schematic target-state profiles, not empirically fitted or universal trajectories.

In summary, in the PAL-D model the director first selects one of the four representative TS types (TS_calm, TS_tension, TS_crisis, TS_relief), or some combination of them, and treats the desired ΔP–ΔI–T pattern for that TS as an intended predictive–affective trajectory at the design stage. The director then specifies an S/F/Ac/Ec profile that supports this trajectory and combines the six levers of camera, movement, framing and composition, light, color, and brightness, editing rhythm and transitions, and the sound environment in order to modulate the four PAL layers and thereby reshape the temporal trajectory of the ΔP–ΔI–AO–T–E loop. In empirical work, observed patterns of ΔP, ΔI, T, and E—estimated from viewers’ physiological measures, gaze, and self-reports—can be compared to the TS assumed at the design stage in order to evaluate how well specific PAL-D hypotheses fit observed viewer trajectories. The overall structure is laid out visually across Figures 1, 2 and Table 1. Figure 1 schematically presents the relation between the director-side PAL-D design grid and the viewer-side PAL model. Figure 2A conceptually organizes TS, S/F/Ac/Ec, and the six perceptual–formal levers as a director-side design configuration that biases the four PAL layers. Figure 2B compares the prototypical ΔP(t) trajectories assumed for the four TS types—calm, tension, crisis, and relief—to illustrate that PAL-D defines TS as ΔP-centered temporal patterns rather than simple emotion labels. Table 1 succinctly summarizes, for each TS type, the hypothesized ΔP–ΔI–T trajectory and the proposed representative S/F/Ac/Ec profile, providing a working coordinate system that can be used in subsequent scene analysis and empirical study design.

## 4 Applications of PAL-D: scene design and research agenda

So far, PAL-D has been developed as a conceptual framework that articulates the relations among TS, S/F/Ac/Ec, the six levers, the four PAL layers, and the ΔP–ΔI–AO–T–E loop. This structure provides a single coordinate system for specifying which variables a director can shape when designing a scene and which aspects of the viewer’s predictive–affective loop can in principle be influenced, estimated, and measured. PAL-D is not a quantitative model that computes exact trajectories, but a mid-level framework for making these design and outcome variables explicit. In practical design and measurement, PAL-D considers the full ΔP–ΔI–AO–T–E loop but focuses on ΔP, ΔI, T, and E as the main design and outcome dimensions, with AO treated as latent as defined in Section 2. The practical value of PAL-D therefore depends on how this coordinate system can be used in scene design, empirical research, directing education, and human–AI collaborative directing, and on whether these uses translate into testable research agendas. This section first examines the tools PAL-D offers for scene design and education and then outlines a research agenda for experimental and observational studies and AI-related applications.

From the perspective of scene design, PAL-D helps transform the intuitive question “What kind of affective pattern, especially what kind of ΔP-centered TS, do I want this scene to create?” into a structured design problem. The director first chooses the TS of a scene as one of the four representative patterns, or as a combination of them, and treats the desired temporal trajectory of ΔP, ΔI, T, and E for that TS as an intended design target. At this stage, TS is closer to a target state vector expressed in PAL-D language than to an empirical description of ΔP–ΔI–T–E patterns observed in viewers; it later serves as a baseline against which empirical data can be compared. For example, early scenes of a mystery drama might be designed to realize a slowly mounting tension TS (TS_tension), with ΔP(t) starting at a low level, gradually rising in the middle, remaining at a medium-to-high level until just before the climax, and delaying full resolution until the scene ends. ΔI(t) is assumed to show a typical lagging pattern, rising slightly later than ΔP(t), and T(t) to shift from near neutrality toward an “about to explode” tense condition. The assumed lag of ΔI relative to ΔP is grounded in general response characteristics of physiological arousal measures such as skin conductance responses, heart rate, and heart-rate variability, but the actual lag, individual differences, and genre-specific differences remain hypotheses for later empirical work. Put differently, a slow-burn TS_tension scene in a detective film or series is one in which viewers feel that “something is wrong and might soon break,” and PAL-D rewrites that intuition as a particular ΔP–ΔI–T trajectory within a specified TS. PAL-D encourages directors to state such goals in predictive–affective terms as “In TS_tension, ΔP rises gradually and remains high; ΔI shows a delayed rise; T converges toward a tense condition; and E is integrated as a tension episode,” while continuing to treat AO as a latent structure projected into these patterns and into narrative understanding and recall.

The next step is to specify the S/F/Ac/Ec profile that supports the chosen TS and then translate this profile into decisions at the level of the six levers. In the TS_tension example, the director might maintain S at a medium-to-high level, keep the overall temporal, spatial, and causal frame reasonably stable, gradually narrow gaze and information onto one or two characters and key clues (increasing F), provide foreshadowing cues while leaving some causal relations partially unexplained (medium Ac), and allow empathy with a particular character to strengthen over time (medium-to-high Ec). Camera position and distance and framing and composition can be adjusted so that the proportion of close-ups around key characters and clues gradually increases, raising F. Light, color, and brightness maintain an everyday tone but increase contrast at key moments to highlight important cues. Editing rhythm starts with relatively longer shots and gradually tightens, and the sound environment moves from predominantly diegetic sound to the gradual addition of low-frequency music and subtle sound effects to support the rise of ΔI.

The same grid can be applied to scenes with different affective goals. Suppose that, in the ending of a feature film, the director aims for a relief TS (TS_relief). ΔP(t) is then assumed to have climbed to a very high level during a preceding crisis TS and to drop sharply within a relatively short interval during the ending sequence, while ΔI(t) decreases more slowly and T(t) turns from tension and anxiety toward a relief-oriented affective condition and converges back toward a stable state. To achieve this, the director can avoid unnecessary ΔP spikes, maintain high Ac so that spatial, temporal, and relational structures are explicitly reintegrated, and regulate F and Ec around moderate levels. Long and medium shots can visually reunify previously fragmented spaces and relationships, while dialogue, action, and possible flashbacks weave scattered cues into a single story, raising Ac and creating conditions for rapid convergence of ΔP. Editing rhythm shifts from the fast and irregular cutting of a crisis TS toward longer, more stable shots, and the sound environment transitions from high-tension music toward more modulated music and sparser ambient sound. Camera movement becomes smoother as abrupt handheld motion gives way to panning and tracking.

These examples show how PAL-D translates intuitive scene goals into ΔP–ΔI–T–E trajectories, S/F/Ac/Ec profiles, and lever choices. In directing education, instructors can move beyond generic guidance such as “Make this part more tense” and instead collaborate with students in PAL-D terms, asking at what point ΔP should begin to rise, how long it should remain elevated, which S level is appropriate, and how Ac, F, and Ec should be managed to sustain a given TS. Students, in turn, can articulate TS, the desired ΔP–ΔI–T–E pattern, and the S/F/Ac/Ec profile before exploring lever combinations. PAL-D is proposed here less as a mandatory classroom toolkit than as an analytical and pedagogical interface that translates intuitive language into predictive–affective structure and formal variables.

From the perspective of empirical research, PAL-D suggests study designs in which TS, S/F/Ac/Ec, and the six levers are treated as independent variables and ΔP, ΔI, T, and E as dependent or mediating variables. ΔP itself corresponds to prediction-error signals at the neural level and is therefore difficult to measure directly, but formal manipulations such as unexpected transitions at event boundaries, mismatches in framing and gaze position across cuts, or sudden omission or addition of narrative information can be used to generate ΔP candidates that are then estimated indirectly via gaze data, self-reports, and, in some cases, measures such as pupillometry or mouse tracking. Eye tracking can reveal changes in fixation patterns and saccades around candidate points of large ΔP, which can be combined with time-series self-reports of surprise and tension. Conversely, successful implementation of TS_calm should result in relatively stable gaze positions across cuts, which can be interpreted as a joint indicator of low ΔP and a stable TS.

ΔI can be quantified using autonomic indices such as skin conductance responses, heart rate, heart-rate variability, and breathing patterns. Skin conductance tends to reflect relatively fast changes, whereas heart-rate variability captures slower regulatory dynamics, suggesting that ΔI is best treated as a multidimensional construct composed of sympathetic activation and parasympathetic regulation rather than reduced to a single index. A plausible agenda is to use quickly responsive indicators such as skin conductance or transient increases in heart rate as primary proxies for ΔI, while collecting HR, HRV, and breathing as secondary indices. T and E can be estimated using time-series self-reports during or immediately after viewing, segment-by-segment ratings of affect and emotion, and measures of narrative memory and recall. T is closer to the unfolding of the dominant affective condition within segments, whereas E denotes the integrated affective condition at the episode level rather than a specific emotion category; which specific emotion this corresponds to lies beyond PAL-D’s scope. Comparing the timing and duration of reported uncertainty, tension, and relief across conditions in which the same narrative material is directed toward different TS types provides an initial test of the ΔP–ΔI–T–E structure assumed by PAL-D.

On this measurement basis, PAL-D suggests several representative research programs. One basic experimental design is to manipulate TS type as an independent variable: keeping narrative content constant, multiple versions of a scene can be created so that each version corresponds to TS_calm, TS_tension, TS_crisis, or TS_relief by altering editing rhythm, camera movement, and sound design, after which temporal patterns of ΔP candidates, ΔI, T, and E can be compared across conditions. Another design manipulates S and Ac to test their influence on the magnitude and persistence of ΔP. Two versions of the same crisis event, one in which ample narrative and contextual cues are provided in advance (high Ac) and another in which key information is withheld (low Ac), can be used to compare peak size and duration of ΔP, subsequent ΔI trajectories, and T and E configurations. Such designs do not aim at precise predictions for individuals but at exploring, in relatively homogeneous groups, the correlation structure between average ΔP–ΔI–T–E patterns and combinations of TS, S/F/Ac/Ec, and levers. Individual, genre-related, and cultural differences are treated as boundary conditions on the applicability of PAL-D and are further considered at the theoretical level in the Discussion and Limitations sections. Taken together, these suggestions amount to a family of testable hypotheses, for example that crisis TS scenes with low initial Ac followed by high Ac will show larger ΔP spikes but faster subsequent convergence than crisis TS scenes with consistently medium Ac, that increases in S while holding Ac constant will increase the number of ΔP candidate points without proportionally increasing the speed of convergence, and that TS_relief implementations will exhibit steeper declines in ΔP and ΔI than TS_calm segments that merely maintain low ΔP levels from the outset.

PAL-D can also support observational studies and text-based analyses. Sequences from existing dramas, films, and entertainment programs can be coded onto the PAL-D grid, and TS transition patterns, S/F/Ac/Ec profiles, and lever combinations can be compared. Scenes that function as a TS_crisis type across different directors’ works, for example, can be grouped to identify typical editing, camera, and sound strategies used to amplify ΔP. Similarly, characteristic S/F/Ac/Ec profiles for TS_tension and recurrent strategies for bringing ΔP and ΔI to convergence in TS_relief can be catalogued. Director commentaries and interviews can be reinterpreted in PAL-D terms: statements such as “Here I wanted the audience to be unable to breathe” or “I wanted them to barely hold on and then finally break down in tears” can be translated into TS types, ΔP–ΔI–T–E patterns, and S/F/Ac/Ec profiles. Such analyses help map intuitive language onto the cognitive–affective structures posited by PAL and PAL-D and generate more concrete hypotheses for subsequent experiments, while providing baseline data on TS and S/F/Ac/Ec patterns across genres, directorial styles, and cultural contexts.

Finally, PAL-D can serve as a theoretical reference point for human–AI collaborative directing and automated editing systems. An algorithm inspired by predictive processing could analyze event structure, gaze distributions, and shooting and editing patterns in input footage to estimate the current TS and S/F/Ac/Ec profile and then provide feedback on how closely the current ΔP–ΔI–T–E structure matches the TS intended by the director. If analysis of viewers’ gaze, physiological signals, and self-reports shows that ΔP and ΔI do not rise sufficiently in a scene intended as TS_tension, the system could propose lever combinations to raise S or to adjust F and Ec, such as more assertive close-ups, more irregular editing rhythms, or more dissonant sound. Conversely, if excessive ΔP spikes are detected in a scene intended as TS_calm, the system could recommend smoothing editing rhythm or adding narrative and contextual cues to increase Ac and attenuate ΔP. For generation rather than diagnosis, a generative video system could treat PAL-D descriptors as scene-level metadata: a prompt could specify a target state together with an S/F/Ac/Ec profile—for example, TS_tension with medium-to-high S, progressively narrowing F, medium Ac, and rising Ec—which the model would attempt to translate into shot scale, camera movement, editing rhythm, sound, and cue density. In a text-to-video or automated-editing workflow, the same metadata could also function as consistency constraints, discouraging abrupt shifts in sensory density, gaze anchoring, or narrative–contextual cueing that would disrupt the intended ΔP–ΔI–T–E pacing across adjacent shots. These scenarios are not immediate design proposals for ready-to-use systems but illustrations of which variables and relations must be exposed to make the question “How should we design the predictive–affective structure of this scene?” tractable in computational form.

Across these applications in scene design and research, PAL-D is consistently treated not as a finished quantitative model but as a testable theoretical framework. The TS–S/F/Ac/Ec–lever–ΔP–ΔI–T–E structure and the TS profiles in Table 1 are, at present, hypothetical coordinate systems rather than established empirical facts. For these coordinate systems to prove useful, actual scene designs and viewing data must repeatedly reveal stable correlation patterns among TS transitions, ΔP–ΔI–T–E trajectories, S/F/Ac/Ec profiles, and lever combinations, while cultural, genre-related, and individual variations and boundary conditions are systematically documented. The role of the present article is not to preempt this validation work but to clarify in theoretical terms how PAL-D can be tested and which variables and relations are primary targets of such testing. On this basis, the following sections summarize the theoretical contributions and limitations of PAL-D and offer a balanced assessment of its potential benefits and risks for media psychology, directing education, and the content industry.

## 5 Discussion

The PAL-D model seeks to connect the director’s design language with the viewer’s predictive–affective structure by re-situating insights from predictive processing, research on emotion and appraisal, event segmentation research, and film and directing theory within a single ΔP-centered coordinate system. Viewer-side PAL provides a cognitive–affective grid that describes what happens inside the viewer through four processing layers and the temporal structure of the ΔP–ΔI–AO–T–E loop. PAL-D adds a thin director-side design grid on top of this structure and asks how particular design choices alter the viewer’s predictive–affective loop, using ΔP-centered TS patterns, S/F/Ac/Ec, and six perceptual–formal levers as its core vocabulary. Its central contribution lies in rewriting a directing language that has long relied on genre intuitions and emotion labels into a structural language of ΔP(t) trajectories and TS–S/F/Ac/Ec–lever combinations, thereby allowing directing intentions and audience responses to be treated within a shared predictive–affective coordinate system. Qualitative terms such as tension, fear, poignancy, and relief remain important, but PAL-D encourages the more precise question of which ΔP–ΔI–T–E trajectories, which S/F/Ac/Ec profiles, and which formal patterns underlie these experiences. PAL-D’s claims fall into three levels, which it is useful to state explicitly. Its core commitments are the theoretical positions on which the framework stands; a set of provisional hypotheses specify likely but revisable regularities; and a set of disconfirmation conditions state what would count against the core commitments. These three levels are summarized in Table 2. These conditions are stated at the level of kinds of outcome rather than specific numerical thresholds, which remain provisional, and would be assessed under the measurement strategies outlined in Section 4—where ΔP is estimated indirectly from gaze and self-report at candidate prediction-error points, ΔI from autonomic indices such as skin conductance, heart rate, and heart-rate variability, and T and E from time-series self-reports, segment ratings, and recall. A failed provisional hypothesis prompts local revision; the disconfirmation conditions bear on the core commitments. This is the sense in which placing ΔP first is predictive: it yields results that can come out against the framework.

**Table 2:** Three levels of commitment in PAL-D.

Level	Content
Core commitments	ΔP is the change in prediction error, not affect; ΔP and T are dissociable rather than synonymous; the predictive–affective process is a recurrent loop, not a one-way chain; a TS is an intended trajectory, distinct from the observed pattern.
Provisional hypotheses	ΔI follows ΔP with a temporal lag; S, F, Ac, and Ec partly co-vary; the four proposed TS patterns are expected to show characteristic ΔP trajectories; higher Ec is expected to amplify ΔI and steepen the gradient of T.
Disconfirmation conditions	Under controlled manipulations of the relevant design axes and with narrative content held fixed, scenes designed as TS_tension/TS_crisis systematically fail to show the predicted rise and sustained elevation of ΔP (and ΔI)—that is, the predicted ordering of trajectories does not appear stably across repeated, controlled manipulations rather than failing in a single scene or on a single index; across the relevant manipulations, ΔP and T show no systematic dissociation; manipulating Ac has no systematic effect on the magnitude or persistence of ΔP.

On the theoretical side, PAL-D reconceptualizes the relations among ΔP, ΔI, AO, T, and E not as a single linear chain but as a differentiated loop structure. Many earlier discussions implicitly assumed a story in which interpretation generates arousal and arousal generates emotion. PAL and PAL-D instead treat AO and ΔI as partially independent processes that co-vary under the joint influence of ΔP and through interactions between lower and higher levels of processing. AO denotes the set of multiple interpretive hypotheses activated in the Appraisal layer, whereas ΔI reflects changes in physiological arousal modulated by prediction error, bodily state, and allocation of attention. These processes do not always form a simple narrative such as “the scene feels threatening, therefore my heart races”; rather, they jointly reflect how ΔP is generated, maintained, and resolved over time. In the Integration layer, the multivalued structure of AO tends to converge into a single dominant affective condition T, and this T combines with ΔI, memory, and self-narrative to form the integrated affective condition E. PAL and PAL-D thus frame emotion not as a one-way path from stimulus to interpretation, arousal, and then emotion, but as a recurrent loop in which ΔP, ΔI, AO, T, and E continuously feed back into one another. In line with the Hypothesis and Theory format, this article clarifies the role and multivalued structure of AO in Section 2, then focuses mainly on the temporal structure of ΔP–ΔI–T–E in developing PAL-D to avoid excessive notational complexity. The structural distinction between AO and T and the assumption that changes in AO distributions mediate the formation of T and E nevertheless remain part of the conceptual backbone supporting PAL-D as a whole.

PAL-D also differs from many existing accounts by treating ΔP(t) as the primary organizing axis of predictive–affective structure and by defining TS not as emotion labels but as ΔP-centered temporal patterns. Treating ΔP(t) as the primary axis does not reduce it to a relabeling of tension, crisis, or relief. ΔP is not affect but the change in prediction error—a formal quantity (the mismatch between predicted and actual input, or surprisal) that predictive processing explicitly distinguishes from surprise in the experientially loaded sense (Clark, 2013). Because of this distinction, ΔP can be high where little tension is felt (a harmless surprise) and low where tension is sustained (a foreseen, slowly approaching threat); ΔP and T are therefore dissociable rather than synonymous. The contribution of placing ΔP first is not to posit a new cause of emotion but to bring heterogeneous formal choices and heterogeneous felt outcomes onto a single, time-resolved coordinate on which they can be compared and from which falsifiable predictions can be derived. In this sense PAL-D’s use of ΔP is integrative and predictive, not explanatory in the sense of positing a novel mechanism. The four proposed representative target-state patterns—calm, tension, crisis, and relief—are distinguished by when and by how much ΔP rises, how long it remains elevated, and when and at what rate it declines, while ΔI and T are treated as associated trajectories that often covary with, and may lag behind, these ΔP patterns. A tension TS is defined by a structure in which ΔP starts low, rises gradually, and remains at a medium or higher level for a certain interval. This characterization is consistent with general psychological models of tension and suspense, in which these states arise from predictive processes oriented toward future, emotionally significant events rather than from arousal as such (Lehne and Koelsch, 2015); PAL-D recasts this viewer-side affective structure as a director-side design target. A crisis TS is characterized by a sharp spike in ΔP over a short interval accompanied by high ΔI and an intense affective condition. A relief TS starts from an elevated ΔP state and is defined by a relatively rapid decline in ΔP over a short period, with ΔI decreasing more slowly with a lag. In PAL-D, TS is not an observed pattern but an intended ΔP–ΔI–T–E trajectory specified at the design level. TS is a target pattern within the director’s design space, and ΔP–ΔI–T–E functions measured in actual viewers provide independent evidence for how closely the realized response matches this target. By keeping intended TS and observed trajectories conceptually separate, PAL-D reduces the risk of circularity—defining TS in terms of ΔP and then “confirming” TS by re-reading ΔP—and positions itself as a comparison framework between design hypotheses and empirical data.

The introduction of the director-side design axes S, F, Ac, and Ec and the six perceptual–formal levers is a distinctive extension beyond viewer-side PAL. S and F are proposed to regulate, at the Gaze/Input layer, how much sensory and informational load must be processed at once and how narrowly or broadly gaze and information are distributed. Ac regulates the richness and coherence of priors and the clarity of temporal, spatial, and causal structure in the Prediction and Appraisal layers, whereas Ec modulates the slope and intensity of ΔI, T, and E in the Appraisal and Integration layers. The six levers are the concrete formal tools that implement these axes, each influencing the four PAL layers in different ways. Analytically, PAL-D treats S, F, Ac, and Ec as independent dimensions, but it explicitly acknowledges that some axes are likely to move together in real-world implementation. Combinations such as high S with high F or low Ac with high Ec may become typical patterns within particular genres, directorial styles, or cultural contexts. Such correlation structures are treated as features of actual implementation. Subsequent empirical work will need to explore how S/F/Ac/Ec in fact cluster together and how the empirical factor structure of these axes aligns with or diverges from the analytical distinctions proposed in PAL-D. In principle these contributions are dissociable: holding shot scale—and thus F—constant while varying only the emotional content of what is framed isolates Ec, whereas holding the empathic content constant while reframing from a tight close-up to a wide shot varies F. PAL-D treats such contrasts as the testable basis for separating the axes, not as separations it has already achieved.

The treatment of Ac and Ec illustrates this stance. In directing practice, a single choice—such as a close-up of an actor—often functions simultaneously as a narrative–contextual cue and as an empathic cue. PAL-D nevertheless defines Ac and Ec as distinct axes to analyze their respective contributions to ΔP, ΔI, T, and E. Ac shapes and revises the structure of priors: when cues about time, space, causality, and relationships are ambiguous or missing, the same input is more likely to generate large and persistent ΔP; when Ac is rich and coherent, even large ΔP spikes can be followed by swift reconstruction of a new model and rapid convergence of prediction error. Crisis TS, where low initial Ac leaves threat and causal gaps unexplained, driving ΔP sharply upward, followed by the introduction of explicit threat and catastrophe cues that increase Ac and support reconstruction of a new model, exemplifies this assumption. Ec, in turn, is expected to amplify the slopes of ΔI, T, and E by modulating how important a given ΔP pattern feels to the viewer. Greater empathy and identification with characters can be associated with a larger increase in ΔI, a stronger affective condition, and a more prolonged affective episode for the same ΔP spike. In this process, Ec not only shapes meaning evaluation in the Appraisal layer but also indirectly influences Prediction-layer strategies about how much ΔP to allow and for how long. PAL-D therefore argues that even if Ac and Ec co-vary in actual scenes, defining them as separate analytical dimensions is necessary if their contributions to ΔP and to ΔI, T, and E are to be manipulated and measured independently.

## 6 Limitations, viewer divergence, and future directions

In terms of scope and limitations, PAL-D is explicitly a mid-level model and does not aspire to explain human cognition or affect in general. Its domain is passive viewing of audiovisual content and, within that domain, the link between the design of individual scenes and sequences and their predictive–affective structure. Macro-level structures of long-form narratives, production environments and platform structures, individual differences, and cultural variation all lie beyond the direct explanatory scope of PAL-D. This delimitation specifies the model’s range of validity. PAL-D does not claim to have empirically separated the contributions of S, F, Ac, and Ec, nor to have specified how they interact; their dissociation and the structure of their interactions remain open empirical questions. Relatedly, the literatures cited throughout support the component assumptions from which PAL-D is assembled—predictive processing, event segmentation, situation-model research, suspense theory, and film emotion theory—but they do not yet validate the specific PAL-D architecture as a whole, including the S/F/Ac/Ec axes, the four TS profiles, and the proposed mappings between formal levers and ΔP–ΔI–T–E trajectories. PAL-D can be combined with higher-level accounts such as narrative theory, industry studies, and cultural analysis and with lower-level accounts such as neural mechanism models, but it does not claim to be a self-contained theory of directing. Moreover, PAL-D is not yet quantitatively validated. It is a conceptual framework that integrates predictive processing, research on emotion and appraisal, event segmentation research, film and directing theory, and viewer-side PAL, and it is presented here, in keeping with the Hypothesis and Theory format, as a reference coordinate system to be used in subsequent experiments, text analyses, and educational research.

A further limitation concerns viewer divergence, especially cases in which viewers consciously resist or subvert a director’s intended trajectory. PAL-D treats TS as an intended predictive–affective trajectory rather than as a guaranteed viewer response. Viewers may bring hyper-specific subcultural priors, genre expertise, or meta-awareness of cinematic tropes that change how the same Ac and Ec cues are predicted and appraised, shifting priors at the Prediction layer and the distribution of appraisal hypotheses at the Appraisal layer, and thereby altering both ΔP and T. For example, a viewer familiar with comedy–horror parody may anticipate a trope designed to generate TS_crisis, so that the same cue yields a smaller or absent ΔP spike and an affective condition closer to amusement or ironic distance than to fear. Such cases do not bypass the PAL-D loop but enter it as differences in prior-weighting and appraisal: PAL-D is prior-relative by construction and frames the divergence as the same loop operating on shifted priors, while making no claim to predict the magnitude or direction of such resistant or ironic readings, which depend on viewer-side priors lying outside the segment-level trajectory it fixes as its object. Empirically, such divergences are best treated as boundary conditions to be measured—by collecting viewer priors, genre familiarity, and trope awareness alongside ΔP candidates, ΔI, T, and E.

The current lack of empirical testing is therefore both a limitation and a starting point for a research program. Section 4 has outlined concrete ways in which TS, S/F/Ac/Ec, the levers, and the ΔP–ΔI–T–E structure can be manipulated and measured. The crucial point is that PAL-D treats these variables as time functions rather than static points. This invites hypotheses not only about whether a given manipulation increases or decreases ΔP, ΔI, T, or E, but also about how quickly and with what slopes ΔI and T follow ΔP spikes in different TS, how long elevated ΔP and ΔI are sustained before convergence, and how convergence speeds in relief TS types relate to the more stable baselines of calm TS types. In this way, the model translates intuitive claims about “slow-burn tension,” “sudden shock,” or “long-delayed catharsis” into time-series predictions that can, in principle, be tested empirically.

In directing education and practice, PAL-D can function as a translational tool between the language of intuition and anecdote and the language of predictive–affective structure and formal variables. The aim is not to replace intuition, but to articulate which ΔP–ΔI–T–E trajectories, S/F/Ac/Ec profiles, and lever combinations intuition in fact relies on, so that these can be shared, critiqued, and refined. In human–AI collaborative directing and automated editing system design, PAL-D can also serve as a theoretical reference: not a tool for automating directing decisions, but a common language for making directing intentions explicit in predictive–affective terms and for structuring feedback about them.

Finally, PAL-D should be understood, together with viewer-side PAL, as part of a broader research program that aims to reframe audiovisual directing and viewing in predictive–affective terms. PAL-D does not replace viewer-side PAL but extends it, taking its cognitive–affective grid as a premise and asking how directors can design predictive–affective loops on that basis. By formalizing PAL-D and discussing its implications for scene design, research agendas, education, and practice, this article has proposed one way of bridging conceptual gaps among predictive processing, research on emotion and appraisal, film and directing theory, and media psychology. In future work, PAL and PAL-D will need to be tested and revised through experimental studies, analyses of real-world content, directing education programs, and human–AI collaborative systems. Through this process, a predictive–affective approach to audiovisual directing—and, more broadly, a research program in what might be called directorial psychology—can take more concrete shape.

## Data Availability

The original contributions presented in the study are included in the article/supplementary material, further inquiries can be directed to the corresponding author.

## References

[ref1] Anderson E. C. Carleton R. N. Diefenbach M. Han P. K. J. (2019). The relationship between uncertainty and affect. Front. Psychol. 10:2504. doi: 10.3389/fpsyg.2019.02504, 31781003 PMC6861361

[ref2] Arnheim R. (1974). Art and Visual Perception: A Psychology of the Creative Eye. Berkeley, CA: University of California Press.

[ref3] Bordwell D. (1985). Narration in the Fiction Film. Madison, WI: University of Wisconsin Press.

[ref4] Carroll N. (1984). Toward a theory of film suspense. Persistence Vision 1, 65–89.

[ref5] Clark A. (2013). Whatever next? Predictive brains, situated agents, and the future of cognitive science. Behav. Brain Sci. 36, 181–204. doi: 10.1017/S0140525X12000477, 23663408

[ref6] Friston K. (2010). The free-energy principle: a unified brain theory? Nat. Rev. Neurosci. 11, 127–138. doi: 10.1038/nrn2787, 20068583

[ref7] Lehne M. Koelsch S. (2015). Toward a general psychological model of tension and suspense. Front. Psychol. 6:79. doi: 10.3389/fpsyg.2015.00079, 25717309 PMC4324075

[ref8] Loschky L. C. Larson A. M. Smith T. J. Magliano J. P. (2020). The scene perception & event comprehension theory (SPECT) applied to visual narratives. Top. Cogn. Sci. 12, 311–351. doi: 10.1111/tops.12455, 31486277 PMC9328418

[ref9] Loschky L. C. Smith M. E. Chandran P. Hutson J. P. Smith T. J. Magliano J. P. (2026). The role of event understanding in guiding attentional selection in real-world scenes: the scene perception & event comprehension theory (SPECT). Atten. Percept. Psychophys. 88:92. doi: 10.3758/s13414-026-03234-7, 41807844 PMC12975865

[ref10] Magliano J. P. Miller J. Zwaan R. A. (2001). Indexing space and time in film understanding. Appl. Cogn. Psychol. 15, 533–545. doi: 10.1002/acp.724

[ref11] Magliano J. P. Zacks J. M. (2011). The impact of continuity editing in narrative film on event segmentation. Cogn. Sci. 35, 1489–1517. doi: 10.1111/j.1551-6709.2011.01202.x, 21972849 PMC3208769

[ref12] Münsterberg H. (1916). The Photoplay: A Psychological Study. New York, NY: D. Appleton.

[ref13] Scherer K. R. (2005). What are emotions? And how can they be measured? Soc. Sci. Inf. 44, 695–729. doi: 10.1177/0539018405058216

[ref14] Scherer K. R. (2009). The dynamic architecture of emotion: evidence for the component process model. Cogn. Emot. 23, 1307–1351. doi: 10.1080/02699930902928969

[ref15] Scherer K. R. Moors A. (2019). The emotion process: event appraisal and component differentiation. Annu. Rev. Psychol. 70, 719–745. doi: 10.1146/annurev-psych-122216-011854, 30110576

[ref16] Seth A. K. Friston K. J. (2016). Active interoceptive inference and the emotional brain. Philos. Trans. R. Soc. B 371:20160007. doi: 10.1098/rstb.2016.0007, 28080966 PMC5062097

[ref18] Smith G. M. (2003). Film Structure and the Emotion System. Cambridge: Cambridge University Press.

[ref17] Smith M. (1995). Engaging Characters: Fiction, Emotion, and the Cinema. Oxford: Clarendon Press.

[ref19] Sobchack V. (1992). The Address of the Eye: A Phenomenology of Film Experience. Princeton, NJ: Princeton University Press.

[ref20] Tan E. S. (1996). Emotion and the Structure of Narrative Film: Film as an Emotion Machine. Mahwah, NJ: Lawrence Erlbaum.

[ref21] Zacks J. M. (2020). Event perception and memory. Annu. Rev. Psychol. 71, 165–191. doi: 10.1146/annurev-psych-010419-051101, 31905113 PMC8679009

[ref22] Zacks J. M. Speer N. K. Swallow K. M. Braver T. S. Reynolds J. R. (2007). Event perception: a mind-brain perspective. Psychol. Bull. 133, 273–293. doi: 10.1037/0033-2909.133.2.273, 17338600 PMC2852534

[ref23] Zwaan R. A. Radvansky G. A. (1998). Situation models in language comprehension and memory. Psychol. Bull. 123, 162–185. doi: 10.1037/0033-2909.123.2.162, 9522683

